# Organic Acids from Roselle (*Hibiscus sabdariffa* L.)—A Brief Review of Its Pharmacological Effects

**DOI:** 10.3390/biomedicines8050100

**Published:** 2020-04-28

**Authors:** Jeannett A. Izquierdo-Vega, Diego A. Arteaga-Badillo, Manuel Sánchez-Gutiérrez, José A. Morales-González, Nancy Vargas-Mendoza, Carlos A. Gómez-Aldapa, Javier Castro-Rosas, Luis Delgado-Olivares, Eduardo Madrigal-Bujaidar, Eduardo Madrigal-Santillán

**Affiliations:** 1 Instituto de Ciencias de la Salud, Universidad Autónoma del Estado de Hidalgo, Ex-Hacienda de la Concepción, Tilcuautla 42080, Mexico; 2 Escuela Superior de Medicina, Instituto Politécnico Nacional, “Unidad Casco de Santo Tomas”, Ciudad de México 11340, Mexico; 3 Instituto de Ciencias Básicas e Ingeniería, Universidad Autónoma del Estado de Hidalgo, Pachuca de Soto 42184, Mexico; 4 Escuela Nacional de Ciencias Biológicas, Instituto Politécnico Nacional, “Unidad Profesional A. López Mateos”, Ciudad de México 07738, Mexico

**Keywords:** citric acid, hibiscus acid, *Hibiscus sabdariffa* L., hydroxycitric acid, malic acid, pharmacological effects, roselle

## Abstract

Roselle (*Hibiscus sabdariffa* L.), also known as jamaica in Spanish, is a perennial plant that grows in tropical and subtropical regions, including China, Egypt, Indonesia, Mexico, Nigeria, Thailand, and Saudi Arabia. It has a long history of uses, mainly focused on culinary, botanical, floral, cosmetic, and medicinal uses. The latter being of great impact due to the diuretic, choleretic, analgesic, antitussive, antihypertensive, antimicrobial, immunomodulatory, hepatoprotective, antioxidant, and anti-cancer effects. These therapeutic properties have been attributed to the bioactive compounds of the plant, mainly phenolic acids, flavonoids, anthocyanins, and organic acids (citric, hydroxycitric, hibiscus, tartaric, malic, and ascorbic). Most literature reviews and meta-analyses on the therapeutic potential of *Hibiscus sabdariffa* L. (Hs) compounds have not adequately addressed the contributions of its organic acids present in the Hs extracts. This review compiles information from published research (in vitro, in vivo, and clinical studies) on demonstrated pharmacological properties of organic acids found in Hs. The intent is to encourage and aid researchers to expand their studies on the pharmacologic and therapeutic effects of Hs to include assessments of the organic acid components.

## 1. Roselle Overview (*Hibiscus sabdariffa* L.)

Roselle, also known as jamaica (in Spanish), red sorrel (in English), or karkadeh (in Arabic), is a perennial plant of the genus Hibiscus (belonging to the Malvaceae family) [1]. It is native to India and Malaysia but because it can grow in marginal soils of low fertility and with low moisture retention, its cultivation has expanded to various tropical and subtropical regions including China, Thailand (these two countries, major global suppliers), Indonesia, Saudi Arabia, Vietnam, Sudan, Egypt, Nigeria, and Mexico [1]. In the specific case of Mexico, approximately 18 thousand hectares are harvested with an average yield of 265 kg. Although the area harvested and the yield per unit area are low, farmers obtain high incomes due to their good level of commercialization, which generates a higher demand of the product [2]. The states of greatest demand are Guerrero and Oaxaca, where approximately 85% of the national production is cultivated. Unfortunately, all the national production comes from a single Creole type variety, which is sown only in the spring–summer agricultural cycle [2].

In general, there are two varieties of jamaica, the first *Hibiscus* var. *Altissima Wester*, cultivated for having a jute-like fiber; and the second, *Hibiscus* var. *sabdariffa*, which presents short and bushy shrubs that have been described in four races: bhagalpuriensi, intermedius, albus and ruber [3]. The most frequently cultivated of them is *Hibiscus* var. *sabdariffa* (Hs) ruber. It is characterized by having a herbaceous shrub, with smooth, cylindrical, and typically red stems. Its leaves are green with lengths that vary between 7.5 and 12.0 cm. Its flowers are up to 5 inches (12.5 cm) wide, yellow and may turn pink when they wilt. Its calyx, stems, and leaves are acidic and have a blueberry-like taste (*Vaccinium spp*.) [3].

## 2. Traditional Uses of Hs

Hs has a long history of uses ranging from China, Egypt, India, Indonesia, Malaysia, Mexico, Thailand, Trinidad and Tobago, Sudan, and some countries in South America [1]. In general, the uses have been focused on culinary, medicinal issues, as a source of cosmetics and on botanical and/or floral aspects [4]. In the culinary case, the fresh or dried calyces and the flower pods of Hs are used for the preparation of hot and cold drinks, tea, fermented beverages, wines, jams, jellies, ice cream, chocolates, aromatic agents, and cakes [1]. Drinks prepared with Hs have been traditionally consumed by different cultures. For example, in Egypt, calyces are used to make “cacody tea” and fermented beverages, while in Sudan and Nigeria, they are boiled with sugar to produce a beverage known as Karkade or Zoborodo. In West India, the calyces are commonly used as a colorant and flavoring for rum. The flower, used in Mexican cuisine, is used in the drink known as jamaica water or jamaica tea, as well as in different typical dishes [1]. There is evidence (in Sudan, Malaysia, China and Africa) that its leaves are ingested raw or cooked, like a vegetable, while the seeds are eaten roasted or ground and used to prepare oils or as a substitute for coffee [4]. Regarding its use as a cosmetic agent, Malaysians often use the oil from their seeds to produce scrubs and soaps [3].

**Figure 1:**
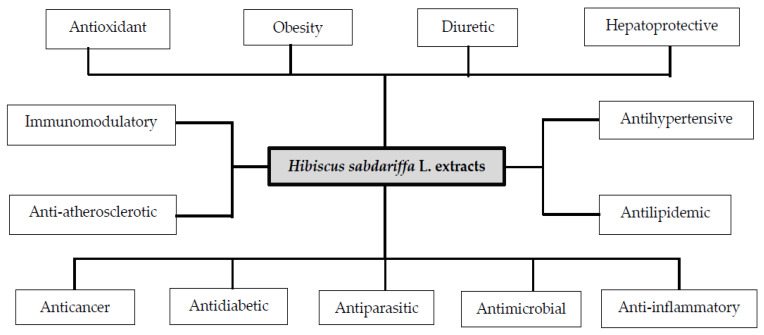
Therapeutic and/or pharmacological properties of *Hibiscus sabdariffa* L. extracts.

However, the greatest impact of Hs has been in the Traditional Medicine/Complementary and Alternative Medicine (TCAM) where it has shown diuretic, choleretic, analgesic, antitussive, and hypotensive effects. Other effects observed are that it lessens blood viscosity, stimulates intestinal peristalsis and reduces body temperature. Likewise, it has been used to treat nervous diseases, cardiovascular diseases and atherosclerosis, obesity, liver disorders, control arterial hypertension, and genital problems [3,5,6,7]. Certain scientific evidence found in the literature confirms its antioxidant, antidiabetic, antilipidemic, antihypertensive, immunomodulatory, hepatoprotective, diuretic, antimicrobial, antiparasitic, and anti-cancer capacities (Figure 1) [1,7,8,9,10,11,12,13]. 

## 3. Nutritional Value and Main Bioactive Compounds of Roselle

In general, the calyx of roselle is red, which gives the traditional color to drinks and infusions prepared with this plant. This characteristic color is attributed to the content of anthocyanins while its acidic taste is due to the content of organic acids such as citric, malic, tartaric acid, and hibiscus [14]. The nutritional composition of Hs has been studied on different occasions and various compounds with nutritional capacity have been found in the plant such as proteins, lipids, vitamins, fiber, amino acids. The percentage and/or quantity of these compounds differ in each variety of jamaica and the anatomical place of the plant that is studied. Thus, the percentage is different in calyces, dried leaves, or even in seeds [15]. Table 1 summarizes the nutritional compounds of each anatomical part of the plant.

The therapeutic properties of *Hibiscus* *sabdariffa* L. (Figure 1) have been attributed to the bioactive compounds (phytochemicals) highlighting phenolic acids (especially protocatechuic acid), flavonoids, anthocyanins (delfinidine-3-sambubioside and cyanidine-3-sambubioside), organic acids, and some polysaccharides, on the whole [3]. The main phytochemicals found in Hs flowers are anthocyanins, flavonoids, organic acids (mostly citric acid, hibiscus acid, and malic acid), glycosides, and fiber. While in calyxes, a similar proportion of these same organic acids and anthocyanins is observed but a low quantity of flavonoids and glycosides [16,17,18,19].

Carvajal-Zarrabal et al. (2012) [16] consider that the available information on the bioactive components of *Hibiscus Sabdariffa* L. (Hs) has increased significantly since 2003. Most of the studies coincide in mentioning that the beneficial effects of Hs (Figure 1) are mainly attributed to anthocyanins, phenolic acids and flavonoids. A minimum group of authors consider organic acids (Hibiscus acid (HA), hydroxycitric acid (HCA), citric acid (CA), malic acid, tartaric acid, and ascorbic acid) to be responsible for some of these effects. Table 2 shows this difference in the number of studies evaluated and the therapeutic properties related to each group of bioactive compounds. In addition, the concentrations of some phytochemicals obtained from the three main types of roselle calyxes extracts are observed.

Diverse phytochemical studies have validated that the roselle calyces are rich in anthocyanins, phenolic acids, flavonoids, and organic acids. The researchers have mainly used an aqueous or organic solvent to extract these bioactive compounds. It is important to comment and remember that one of the most common uses of roselle calyxes is in the preparation of hot and cold drinks, tea, fermented beverages. Possibly, this is the reason why aqueous extracts are more studied and analyzed.

## 4. Phenolic Acids and Flavonoids

Roselle calyxes are an abundant and interesting source of bioactive molecules such as polyphenols and flavonoids, which have shown antioxidant, hypocholesterolemic, antihypertensive, antimicrobial, anti-inflammatory, anti-diabetic, and anti-cancer potential [4]. In general, Hs contains flavonol and flavanol polyphenols in simple or polymerized form [3]. In some studies, the following flavonoids have been detected: hibiscitrin (hibiscetin-3-glucoside), sabdaritrine, gossypitrin, gossytrin and other glycosides of gossypetin, quercetin and luteolin; as well as chlorogenic acid, protocatechuic acid, pelargonidic acid, eugenol, and sterols (mainly beta-sitosterol and ergosterol) [20,56]. In summary, some evidence about the therapeutic properties of the mentioned compounds are:

(a) Titilayo Fakeye (2008) evaluated the immunomodulatory activity of two fractions (one soluble in residual water and another in ethyl acetate) of an alcoholic-aqueous extract from the dried calyces of Hs in experimental animals. Their results indicated that both fractions significantly increase the production of IL-10 and decrease the induction of tumor necrosis factor alpha (TNF-α) [21]. Possibly this motivated Kao et al., (2009) to analyze in vivo and in vitro the anti-inflammatory effect of some polyphenols extracted from Hs (mainly catechins, protocatechuic acid and caffeic acid). At the end of their investigations, they confirmed that this activity is related to the induction of cyclooxygenase-2 by the negative regulation of the c-Jun N-terminal kinase (JNK) and protein 38 mitogen-activated kinases paths (p38 MAPK) [22].

(b) Subsequently, the protective effect of two doses (250 and 500 mg/kg) of an aqueous extract of Hs was evaluated against the damage caused by potassium bromide in Wistar rats; which were treated with both compounds for 14 days where lipoperoxidation and malondialdehyde levels (MDA) were determined. In addition, the weight of several organs (brain, kidney, stomach, spleen, heart, and liver) of which data were taken as indicators of inflammation and necrosis to be quantified. The results indicated that there was no relevant difference in the proportion of the weight of the organs analyzed in any dose; while the level of MDA was significantly reduced in a dose-dependent manner [23].

(c) Information collected by Da-Costa-Rocha et al., (2014) [3] suggested that roselle’s anti-pyrogenic and anti-inflammatory effects are carried out by a different mechanism from that of NSAIDs, since they observed that ethanolic and aqueous extracts of their calyxes have immunomodulatory function, decreasing the production of proinflammatory cytosines such as TNF-α and IL-6. Another evidence from their studies showed that protocatechuic acid (at an approximate concentration of 5 mg/mL) inhibited the growth of *Staphylococcus aureus*, *Klebsiella pneumoniae*, *Pseudomonas aeruginosa,* and *Acinetobacter baumannii*.

(d) Another property suggested of protocatechuic acid has been as an anti-cancer agent due to its ability to reduce reactive oxygen species (ROS), decrease DNA fragmentation, stop the G1 phase cell cycle and induce apoptosis; being the latter associated with the phosphorylation and degradation of the RB gene (in retinoblastoma) and the suppression of the Bcl-2 protein. Similar effects were observed in human gastric carcinoma (AGS) cells in which the apoptotic induction was suggested to be mediated by p53 gene signaling and the p38 MAPK/FasL cascade pathway [24,57].

(e) The aqueous extracts have been the most studied so far, and this is where the genoprotective effect of some phenolic and flavonoid acids of jamaica has been achieved. With the purpose to determine the antimutagenic potential of an aqueous extract of *Hibiscus sabdariffa* L., Brazilian researchers (2016) [8] conducted an in vivo study. They used Wistar rats that were treated with the extract (400 mg/kg) for 15 days and subsequently a single dose of cyclophosphamide (25 mg/kg). At the end of this period, they evaluated the frequency of micronucleus (MN) in bone marrow; concluding that the extract can reduce its number. In general, these data suggested that the chemopreventive capacity of the Hs extract is related to the presence of flavonoids in their chemical composition.

### Anthocyanins

There are innumerable studies related to anthocyanins where their therapeutic, pharmacological, and/or protective properties have been confirmed; this is the reason why it would be impossible to mention them all. Besides, the majority of studies are on anthocyanins extracted from blueberries, blackberries and prickly pears. Therefore, only brief information will be included in this work.

The first anthocyanin of the Hs calyx that was isolated was “hibycin” (also called delphinidin-3-sambubioside, cyanidine-3-glucoside and/or delphinidin pentoside-glycoside) [58]. Four types of anthocyanins are currently known in the calyxes of jamaica; in greater proportion, delfinidine-3-sambubioside (D3S) and cyanidine-3-sambubioside (C3S), while in lesser relevance delfinidine-3-glucoside and cyanidine-3-glucoside [4]. Of these, 85% of the total anthocyanins is D3S, being considered the main source of antioxidant capacity of *Hibiscus sabdariffa* L. extracts [3].

In general, anthocyanins, particularly D3S and C3S, are believed to be the active components responsible for the antihypertensive and hypocholesterolemic effects of HS, possibly because they are found in large amounts in aqueous extracts [19]. On the other hand, D3S has also been linked to anticarcinogenic activities since it has shown this ability to stimulate apoptosis in neoplastic leukemia cells through the p38-FasL and Bid pathway [25].

## 5. Pharmacological Evidence of Hs Organic Acids

**Figure 2:**
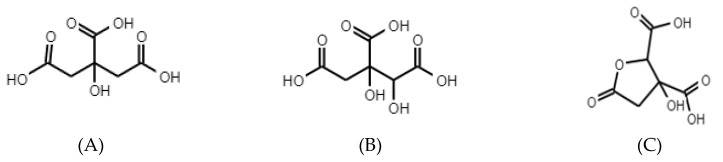
Chemical structure of the main organic acids of Hs. (**A**) Citric acid, (**B**) Hydroxycitric acid, and (**C**) Hibiscus acid [3,16].

Little attention has been paid to the organic acids of *Hibiscus sabdariffa* L., especially to hibiscus acid [59]. The few studies that exist on Hs extracts indicate that they contain a high percentage of organic acids, including citric acid, hydroxycitric acid, hibiscus acid, malic acid, and tartaric acid as the main compounds (the chemical structure of some of these organic acids is shown in Figure 2). While in a smaller proportion oxalic and ascorbic acids have been found, in general, the percentage of these organic acids is similar in the species of *Hibiscus sabdariffa* L., although the hibiscus acid is the most representative (13–24%). The rest of the organic acids are: (a) 12-20% citric acid, (b) 2–9% malic acid, (c) 8% tartaric acid and (d) ascorbic acid between 0.02 and 0.05% [4].

### 5.1. Citric Acid

Citric acid ((CA), chemically known as 2-hydroxy-1,2,3-propanotricarboxylic acid)) was first isolated by the British Karls Scheels (1874), from lemon juice imported from Italy. Due to Italian entrepreneurs who monopolized the production of lemon for almost 100 years and sold it at a high cost, numerous efforts began throughout the world to find alternatives that included chemical and microbial techniques. Among these techniques, the Wehmer process (1923) stands out, who obtained CA as a byproduct of calcium oxalate produced by a culture of *Penicillium glaucum*, as well as its isolation from two varieties of fungi belonging to the *Citromyces* genus. However, these industrial tests were unsuccessful due to contamination problems and to the long duration of the fermentation. Therefore, the industrial process was first opened by Currie in 1917, who discovered that *Aspergillus niger* had the ability to accumulate significant amounts of CA in a sugar-based medium. [60,61].

Citric acid is considered a versatile organic carboxylic acid and widely used in the food industry (for its pleasant acid taste and high water solubility), pharmaceutical, and cosmetic (Table 3) [62]. It is accepted worldwide as a safe agent and has been approved by the Joint FAO/WHO Expert Committee on Food Additives. The pharmaceutical and cosmetic industry retain 10% of its use and the rest is used for other purposes [61,63]. In general, CA is in monohydrated and anhydrous forms. The first [known as CA-E330 (food additive E330)] usually occurs in granulated powder and/or small white crystals that are soluble in water, ether, and ethanol. On the other hand, the anhydrous CA has a different structure since it is free of water, that is, totally dehydrated. However, it usually shows similar properties with the peculiarity that it can capture water with great ease. In both cases, if consumed excessively they are related to irritation of mucous membranes and respiratory tract [63].

It is curious that, although the first isolation took place in 1874 and that it has demonstrated its significant uses in the food, pharmaceutical and cosmetic industries, there are no scientific studies of CA extracted from *Hibiscus sabdariffa* L. Virtually all the evidence on its actions beneficial and/or pharmacological in the medical and health area have been obtained from the extraction of the main citrus species; that is, *Citrus aurantium* (popularly known as bitter orange), *Citrus sinensis* (also known as sweet orange), *Citrus bergamia* (commonly known as bergamot) and *Citrus lemon*. This last group has the lemon as its representative fruit, which is considered the third most important species of the *Citrus* genus, since it contains many relevant natural chemical compounds, such as minerals, ascorbic acid, flavonoids and unquestionably, citric acid [64,65].

In summary, citric acid has shown the following health benefits:

(a) Improves the bioavailability of minerals, allowing the body to absorb them better. For example, calcium citrate does not require stomach acid for absorption and has fewer side effects than calcium carbonate (antacid); making it a good option for people with less stomach acid such as older adults, reducing gas, and constipation. It has also been observed that citrate magnesium is better absorbed and has greater bioavailability than magnesium oxide and magnesium sulfate. Similar absorption occurs with zinc supplements [63].

(b) In the form of potassium citrate, it prevents the formation of kidney stones by making urine less favorable for its formation in the kidneys. That is why eating foods rich in this natural acid, such as citrus fruits, can favor this preventive action [66].

(c) Certain studies have suggested that CA may have important anti-inflammatory and antioxidant effects, so it may help regulate oxidative stress. CA can decrease lipid peroxidation and inflammation by reducing cell degranulation and attenuating the release of inflammatory compounds such as myeloperoxidase, elastase, interleukin, and platelet factor 4 [46]. Likewise, studies conducted by Abdel-Salam et al. (2014) [47], evaluated the effect of this weak organic acid on oxidative stress induced by endotoxins of the brain and liver in Swiss male albino mice. A single intraperitoneal dose of lipopolysaccharide (LPS; 200 µg/kg) was sufficient to induce oxidative stress in both organs, with a consequential reduction of the activity of glutathione, glutathione peroxidase (GPx) and paraoxonase 1 (PON1). Also, the same single dose of endotoxin increased lipid peroxidation (malondialdehyde (MDA)) and TNF-α in brain tissue after the injection. On the other hand, the administration of CA (doses between 1 and 2 g/kg) decreased peroxidation and inflammation of brain lipids, liver damage, and DNA fragmentation. Due to its antioxidant capacity, this alpha hydroxy acid is added to some products (such as serums, masks, and night creams) for skin care in order to adjust the acidity, avoid environmental damage and protect it against photoaging [48].

(d) Finally, it has been suggested that CA may improve the functions of the endothelium; this, when considering that it is a thin membrane that covers the interior of the heart and blood vessels and helps vascular relaxation and contraction, blood clotting, immune function, and platelet aggregation. Therefore, citric acid salts can be used as anticoagulants due to their chelating capabilities of calcium and to reduce inflammatory markers [49].

### 5.2. Hydroxycitric Acid

Hydroxycitric acid (HCA) is a derivative of citric acid found in a variety of tropical plants, including *Garcinia cambogia* and *Hibiscus sabdariffa* L. (Hs). Together with hibiscus acid (HA) they are the main organic acids extracted from the calyxes of Hs. However, *Garcinia cambogia* plants are considered to have the highest amount of HCA [67]. This is probably the main reason why most of the studies in the literature have been carried out with HCA extracted from *Garcinia cambogia*. It is important to note that there are four isomers ((+) and (−)-HCA), and ((+) and (−)-alhydroxycitric acid), the isomer (−)-HCA being found in both plants; also, it is very similar, for the absolute chemical configurations are (2S, 3S in *Garcinia cambogia*) and (2S, 3R in *Hibiscus sabdariffa* L.); thus, there is a high possibility that they have similar pharmacological and/or therapeutic profiles [4,26].

Most scientific evidence of its therapeutic potential focuses on the ability to promote weight loss, suppress de novo fatty acid synthesis, and increase lipid oxidation and/or glycogen synthesis rate [68]. Despite the fact that *H. sabdariffa* calyx extracts (aqueous, ethanolic and methanolic) have shown a wide range of therapeutic effects and that most evidence indicate that they are safe (In general, have an average lethal dose (LD_50_) above 5000 mg/kg, basically evaluated in rodents. There are some conflicting data that extracts can produce adverse effects (mainly related to sperm morphology and liver and kidney function disturbances) in excessive doses and for relatively long periods [5,17,27,28,29,30,31]. Probably, these adverse effects can also be attributed to the presence of their bioactive compounds. In the specific case of HCA (as an isolated and purified compound), Table 4 shows the main studies of its therapeutic actions and its possible toxic effects (especially those related to its irritative capacity in skin and eyes, and its effect on erythropoiesis and fetal development).

### 5.3. Hibiscus Acid

As mentioned above, there is little research related to organic acids extracted from Hs, especially those focused on hibiscus acid (HA). Probably, this scarce scientific attention is attributed to the fact that hibiscus acid is not commercially available and is a chiral compound and diastereomer of garcinia acid (extracted from *Garcinia cambogia*); which is commercially available. Practically, the most representative evidence on their extraction, properties and/or chemical characteristics have been analyzed by Zheoat et al., (2019) [37] and Portillo-Torres et al., (2019) [69]; who mention that their empirical formula is C6H6O7•H2O. Through crystallographic analysis and X-ray spectroscopy confirmed that HA is a five-membered lactone ring (similar to HCA), with four carbon atoms and one oxygen atom. C3 (sp2) has a double-bonded oxygen atom, C1 an OH group and a COOH group, and C2 a COOH group, respectively (Figure 2).

In relation to its therapeutic and/or pharmacological properties, some studies suggest that HA has an inhibitory effect against α-amylase and α-glucosidase; as well as a possible antihypertensive potential. In the first case, Hansawasdi et al., (2000,2001) [38,39] isolated Hibiscus acid and its 6-methyl ester from methanolic and acetonic extracts of roselle, which showed a high inhibitory activity against porcine pancreatic α-amylase. This effect turned out to be more significant when compared to citric acid, which is a known inhibitor of fungal α-amylase. Subsequently, these same compounds were administered in a Caco-2 system supplemented with alpha-amylase where a slight inhibitory effect of α-glucosidase was confirmed in the digestion of starch. At analyzing these results and considering that α-glucosidase is an enzyme located in the intestinal lumen (specifically in the brush border cells), possibly, the suppression of this enzyme decreases the absorption of exogenous glucose affecting postprandial glucose levels. Therefore, HA could be considered an agent with a mechanism of action similar to that of some drugs used in type 2 diabetes, such as acarbose.

Regarding its antihypertensive effect, Zheoat et al., (2019) [37] conducted a comparative study between a crude extract of *Hibiscus sabdariffa* and the HA derived from the same extract on the direct vasorelaxant effect in the Sprague-Dawley rat aorta. At the end of the study, they confirmed that HA was more potent and effective, attributing its vasorelaxant action to the inhibition of Ca^2+^ influx via voltage-dependent Ca^2+^ channels (an identical mechanism observed in garcinia acid; Ha diastereoisomer).

Combining the previous evidence, that is, its antihypertensive capacity and its inhibitory effect of enzymes (α-amylase/α-glucosidase) that block the absorption of sugars and starch and contribute to the loss of body weight, some researchers consider HA as an alternative to prevent some diseases such as dyslipidemia, arterial hypertension, atherosclerosis, acute myocardial infarction, chronic kidney disease, diabetes and metabolic syndrome [36,38].

Recently, researchers from the Autonomous University of the State of Hidalgo in Mexico, explored two new properties of HA. The first one is related to the antimicrobial capacity of 25 isolated fractions of acetonic extracts of the *H. sabdariffa* calyxes containing purified hibiscus acid (HA) and some of its derivatives (monoester and methyl diester, monoester and ethyl diester). The gel diffusion technique found that most of the fractions inhibited the growth of strains of *Escherichia coli*, *Salmonella typhimurium*, *Pseudomonas aeruginosa*, *Staphylococcus aureus*, and *Vibrio cholerae*. The authors concluded that the microorganism most sensitive to inhibition was *Vibrio cholerae*. [69]. Finally, through the micronucleus assay, the genotoxic potential of HA obtained from a methanolic Hs extract in CD-1 mice was evaluated. Initially, its lethal dose 50 was determined, where there was no mortality up to a dose of 5000 mg/kg. With this datum, three doses of HA (250, 500 and 1000 mg/kg) were administered orally for 5 days. Subsequently, at different times (0, 24, 48, 72, and 96 h) blood smears were performed and the number of micronucleated normochromatic erythrocytes (MNNE) and the ratio of polychromatic erythrocytes (PE) to normochromic erythrocytes (NE) were quantified. The results indicated that HA does not increase the frequency of MNNE or alter the PE/NE ratio (cytoxicity index) at any dose or evaluation schedule. These results suggest that HA is not a clastogenic or cytotoxic agent, opening the possibility of exploring its antigenotoxic capacity against different mutagens and/or carcinogens [70].

### 5.4. Tartaric, Malic, and Ascorbic Acids

Tartaric acid (TA) is a well-known organic acid that is found naturally in many fruits, especially grapes. Depending on the property to rotate the plane of polarized light, there are two enantiomers, L (+)-TA and D (−)-TA, the latter being the one that rarely exists in natural sources. The levogyre form (L (+)-TA) is widely used in the food and chemical industry and for the production of wine (main acidity corrector); while the dextrogyre form (D (−)-TA) is more important in the manufacture of pharmaceutical products. Traditionally, TA is obtained as a solid byproduct during wine fermentation, and this production method is strongly influenced by the growth of grapes and climatic conditions. To date, therapeutic and/or pharmacological uses have not been attributed to it; its main use is as a natural acidifying and preservative [52].

Malic acid (MA) is an alpha-hydroxy acid found in various fruits and vegetables. It is frequently used in the cosmetic industry as an exfoliating agent and for the treatment of damaged or dry skin; as well as to control acne. In recent decades, new therapeutic properties have been sought, which is why Gómez-Moreno et al., (2013) [50] evaluated the clinical effectiveness of MA in the form of 1.0% aerosol for the treatment of xerostomy induced by antihypertensive drugs. Their study was a double-blind type consisting of 45 patients, of which 25 were treated with 1.0% malic acid and the rest only with a placebo. Their results indicated that 1.0% malic acid aerosol improved xerostomy induced by antihypertensives and stimulated saliva production. Despite its constant use in cosmetology, interestingly, there are few reports on its skin safety. Therefore, Jen-Hung Yang et al., (2015) [51] analyzed the cytotoxic potential and apoptotic effect of MA in human keratinocyte cell lines (HaCaT). Their results showed that MA induced apoptosis (increased the expression of FasL, Bax, Bid, caspases-3, -8, -9), DNA fragmentation, and the sub-G1 phase in those cells. A flow cytometry analysis also showed that the mitochondrial superoxide production was increased and the mitochondrial membrane potential decreased. They concluded that this organic acid has a significant antiproliferative effect on HaCaT cells by inhibiting the progression of the cell cycle in G0/G1 and stimulating the induction of programmed cell death.

Finally, ascorbic acid can be found in different fruits and vegetables. It is known that most animals can synthesize it from glucose in the liver and intestine, favoring the adequate maintenance of their physiological levels. Unfortunately, humans lack the enzyme S-gulonolactone oxidase, which is essential for its synthesis; deficiency that makes them dependent on exogenous sources of ascorbic acid. It is essential for a wide variety of microorganisms [53,54]. Ascorbic acid (reduced form of vitamin C) is a relevant antioxidant agent of the central nervous system; making it an important compound for brain activity. It is released from the glial deposits to the synaptic cleft and is subsequently absorbed by neurons. In neurons, ascorbic acid favors the elimination of reactive oxygen species (ROS) generated during synaptic activity and neuronal metabolism. Finally, it oxidizes to form dehydroascorbic acid, which is released into the extracellular space, where it can be recycled by astrocytes. Studies have shown that a high level of oxidative stress is generated during neurodegenerative diseases, which induces the constant consumption of available ascorbic acid in the brain. Therefore, if ascorbic acid is used as an antioxidant agent, it oxidizes; and unfortunately, during high levels of ROS its available levels can be decreased. In conclusion, the oxidative stress, high ROS production and the failure of homeostatic systems for the recycling of ascorbic acid are relevant aspects for the progression of neurodegeneration [55].

After conducting an exhaustive review of the different electronic databases, no research was found where these three organic acids extracted from *Hibiscus sabdariffa* L. were used or analyzed, confirming that most of the investigations have focused on considering protocatechuic acid and anthocyanins as the main phytochemicals responsible for the therapeutic and/or pharmacological properties (Figure 1) of the extracts of this plant. However, it is convenient to comment that the combination and/or set of these bioactive compounds in the extracts promote its benefits. Reasons why it would be important to isolate and purify these organic acids from *Hibiscus sabdariffa* L., to evaluate their pharmacological capabilities individually, which opens a field of exploration to new studies that analyze other possible properties to add them to those that have already been confirmed.

## 6. Perspectives and Conclusions

The scientific evidence shown in this manuscript confirms that the organic acids present in *Hibiscus sabdariffa* L. (Hs) may have important therapeutic and/or pharmacological effects for humans. Unfortunately, when analyzing the number of studies, the low impact that the scientific community has shown at exploring these properties is also confirmed. In summary, we can observe the following:

(a) Although citric acid (CA) was isolated since 1874 and is used in the food, pharmaceutical and cosmetic industries, interestingly, there are no studies where CA extracted from Hs has been used. So far, there are 5 relevant investigations (with CA obtained from citrus fruits) that suggest its ability to prevent kidney stone formation and its anti-inflammatory, anticoagulant and antioxidant potential.

(b) Regarding hydroxycitric acid (HCA), most of its therapeutic properties are oriented to its ability to promote weight loss, suppress fatty acid synthesis, and increase lipid oxidation. Such evidence has been observed, mainly in HCA extracted from *Garcinia cambogia*. However, roselle HCA has a similar chemical structure, which considers the question and/or hypothesis that they probably have similar pharmacological profiles. As shown in Table 4, there are 8 studies that suggest these properties and there is only one investigation where this acid extracted from Hs was analyzed.

(c) As mentioned before in this manuscript, the low scientific interest towards hibiscus acid (HA) obtained from roselle might be due to its comercial unavailability; unlike its main diastereomer, the garcinia acid obtained from *Garcinia cambogia* (same plant where HCA is obtained). However, the four investigations that are published so far suggest an antimicrobial, antihypertensive, and enzyme inhibitory effect (α-amylase and α-glucosidase). For this reason, if we combine especially these last two properties, HA could be considered as an alternative to prevent some diseases (such as dyslipidemia, arterial hypertension, atherosclerosis, and diabetes), opening a field of research to new preclinical and clinical studies that confirm its ability to reduce the absorption of sugars, contribute to the loss of body weight and evaluate its vasorelaxant action. In addition, it would be convenient to consider that *H. sabdariffa* extracts (aqueous, ethanolics, and methanolics) have shown anti-cancer potential against different toxic agents in experimental animals and cell cultures (mainly in leukemic cells (K-562), breast adenocarcinoma human (MCF-7), fetal foreskin fibroblasts (HFFF), human gastric carcinoma cells (AGS), human prostate cancer cells (CaP), multiple myeloma cells (RPMI 8226) and oral squamous cell carcinoma cells (SCC-25)). Chemopreventive property that has focused on its ability to inhibit metastasis and induce cytotoxicity and/or apoptosis through different signaling pathways and routes (highlighting: p53, JNK/p38 MAPK/FasL, ERK1/2, caspase 9 mediated by Bax c, Fas 8/t-Bid) and has been mainly related to protocatechuic acid and anthocyanins (specifically, delfinidine-3-sambubioside). [1,4,13,16,17,32,33,34,35,57] However, the chemopreventative mechanisms of action are diverse [71], therefore a field of research is opened to other bioactive compounds of *H. sabdariffa*; in particular, HA showed an LD_50_ greater than 5000 mg/kg in a recent study [70] and presents a chemical structure that suggests exploring its antioxidant and/or antigenotoxic potential against different mutagens and/or carcinogens.

(d) Additionally, after conducting a search in the main electronic databases, no research was found where citric, tartaric, malic, or ascorbic acid extracted from *Hibiscus sabdariffa* L. were used or analyzed. On the contrary, it was confirmed that most of the studies have focused on consider to protocatechuic acid and anthocyanins as the main phytochemicals responsible for the therapeutic and/or pharmacological properties of the extracts of this plant. So it would be convenient to carry out new investigations with these organic acids that provides more information about its beneficial potentials for the human.

Finally, it would be important to develop new investigations with these organic acids individually exploring its capabilities and pharmacological properties, their doses and intervals of administration and analyzing their possible toxic effects in the medium and long term. Therefore, bioactive compounds extracted from Hs could be used in the preparation of pharmaceuticals and nutraceuticals, as well as to obtain chemopreventive agents directed at cancer and/or chronic degenerative diseases. Future studies on Hs extracts and isolated bioactive compounds (including organic acids) will permit an increased knowledge of the beneficial properties of *Hibiscus sabdariffa* L., which is a plant frequently consumed throughout the world and is apparently considered safe.

## Figures and Tables

**Table 1:** Nutritional compounds of *Hibiscus sabdariffa* L.

Nutritional Compound	Calyxes (mg)	Fresh Leaves (%)	Seed (%)
Humidity	9.2	26.2	12.9
Proteins	1.145	1.7–3.2	3.29
Fat	2.61	1.1	
Carbohydrates		10	
Fatty oil			12.9
Celullose			12.9
Pentoses			15.8
Starch			11.1
Fiber	12		
Calcium	1.263	0.18	
Phosphorus	273.2	0.04	
Iron	8.98	0.0054	
Thiamine	0.117		
Riboflavin	0.277		
Niacin	3.765		
Ascorbic acid	6.7		
Malic acid		1.25	

Nutritional compounds in 100 g of roselle [15].

**Table 2:** Therapeutic effects and concentrations of some bioactive compounds from extracts of *Hibiscus sabdariffa* L. calyxes.

Extract Type	Bioactive Compound	Concentrations	Therapeutic Effect Evaluated
**Anthocyanins**			
Aqueus	Delphinidin-3-sambubioside	2701.21 ± 165.55 ppm	Antioxidant, anti-inflammatory, antidiabetic, antiparasitic, antimicrobial, anti-cancer, apoptosis, anti-atherosclerotic, antilipidemic, antihypertensive, obesity, diuretic, hepatoprotective, antimutagenic and immunomodulatory [1,2,3,4,5,6,7,8,9,10,11,12,13,16,17,18,19,20,21,22,23,24,25,26,27,28,29,30,31,32,33,34,35]
	Delphinidin-3-sambubioside	0.78 mg/g	
Ethanolic	Delphinidin-3-sambubioside	7.03 ± 0.04 mg/g	
Aqueous	Cyanidine-3-sambubioside	1939.15 ± 39.27 ppm	
	Cyanidine-3-sambubioside	0.46 mg/mL	
Ethanolic	Cyanidine-3-sambubioside	4.40 ± 0.02 mg/mL	
**Phenolic acids and Flavonoids**			
Aqueous	Chlorogenic acid	1923.72 ± 38.69 ppm	Antioxidant, anti-inflammatory, antidiabetic, antiparasitic, antimicrobial, anti-cancer, apoptosis, anti-atherosclerotic, antilipidemic, antihypertensive, obesity, diuretic, hepatoprotective, antimutagenic and immunomodulatory [1,2,3,4,5,6,7,8,9,10,11,12,13,16,17,18,19,20,21,22,23,24,25,26,27,28,29,30,31,32,33,34,35]
	Chlorogenic acid isomer I	2755.15 ± 62.42 ppm	
	Chlorogenic acid isomer II	1041.19 ± 16.96 ppm	
Aqueous	5-O-Caffeoyl-shikimic acid	171.47 ± 6.92 ppm	
	3-Caffeoylquinic acid	0.36 mg/mL	
Ethanolic	3-Caffeoylquinic acid	2.6 mg/g	
Aqueous	5-Caffeoylquinic acid	0.30 mg/mL	
Ethanolic	5-Caffeoylquinic acid	1.53 ± 0.06 mg/g	
Aqueous	4-Caffeoylquinic acid	1.44 ± 0.08 mg/g	
	4-Caffeoylquinic acid	1.00 ± 0.02 mg/g	
Methanolic	Caffeic acid	18.24%	
	Protocatechuic acid	8.62%	
Aqueous	Quercetin	121.24 ± 2.01 ppm	
	Quercetin-3-sambubioside	304.02 ± 5.90 ppm	
Aqueous	Quercetin-3-rutinoside	495.76 ± 4.34 ppm	
Ethanolic	Quercetin-3-rutinoside	1.07 ± 0.1 mg/g	
Aqueous	Quercitin-3-glucoside	143.74 ± 2.16 ppm	
Ethanolic	Quercetin-pentosylhexoside	1.031 ± 0.002 mg/g	
Ethanolic	Myricetin-pentosylhexoside	0.961 ± 0.001 mg/g	
**Organic Acids**			
Aqueous	Hibiscus acid (HA)	31122.02 ± 1128.39 ppm (13–24%).	Antimicrobial, antidiabetic antihypertensive/vasorelaxant, [36,37,38,39]
	Hydroxycitric acid (HCA)	8288.03 ± 397.63 ppm	Antioxidant, anti-inflammatory, obesity, neuroprotective agent, antilipidemic, [36,40,41,42,43,44,45]
	Citric acid (CA)	12–20%	Anticoagulant, antioxidant, anti-inflammatory [46,47,48,49]
	Malic acid	2–9%	Exfoliating agent, apoptosis, treatment of xerostomy, antiproliferative agent [50,51]
	Tartaric acid	8%	Natural acidifying [52]
	Ascorbic acid	0.02–0.05%	Antioxidant [53,54,55]

**Table 3:** Main applications of citric acid.

Industry	Applications
Canned and/or preserved foods	From fruits and vegetables (lettuce, carrot, celery, spinach, paprika, mushrooms) to beef, chicken and fish; CA monohydrate (food additive E330) lowers the pH, acts as a chelator and prevents enzymatic oxidation avoiding the degradation of food color and taste
Dairy products	CA-E330 is an important stabilizer of whipped creams to maintain their texture. It is an acidifying agent in many cheese products and as an antioxidant.
Cake shop	The main function of the CA-E330 is acidulant. In general, it acts on the conservation, viscosity, and acidity of gels, cakes and/or cupcakes
Beverages	It provides acidity and complements the flavors of fruits and berries. Increases the effectiveness of antimicrobial preservatives and adjusts the pH to maintain a uniform acidity.
Jellies, jams, and preserves	Provides acidity and regulates the pH of the products
Pharmaceuticals	As effervescent in powders and tablets in combination with bicarbonates. Provides rapid dissolution of active ingredients. Acidulant in mild astringent formulation. Anticoagulant.
Cosmetics and toiletries	pH adjustment, antioxidant as a metallic-ion chelator, buffering agent.
Metal cleaning	Cleans and removes iron and copper oxides from surfaces of ferrous and nonferrous metals
Others	Also used in the copper plating, leather tanning, printing inks, bottle washing compounds, textiles, photographic reagents, moulds, adhesives, polymers, and waste treatment

**Table 4:** Main therapeutic evidence of Hydroxycitric acid.

Year	Authors	Main Objective, Results, and Conclusion	References
2003	Hayamizu et al.	Evaluation of oral administration of *G. cambogia* (containing 1000 mg of HCA per day) on the accumulation of visceral fat. It was a 12-week double-blind, randomized, placebo-controlled trial. The evaluation parameters were body indexes (such as height, body weight, waist-hip ratio, and body mass index (BMI)) as well as laboratory test analysis (total cholesterol and free fatty acids). At the end of the study, a significant reduction in abdominal fat was confirmed; regardless of the gender of the patients. There were no significant differences in BMI, only a slight decrease in body weight in men.	[40]
2007	Preuss et al.	The effect of HCA alone and combined with chromium plus niacin (NBC) on weight loss was analyzed in this clinical study. After 8 weeks of administering the compounds to 60 moderately obese subjects, a significant decrease in body weight, BMI and total cholesterol concentrations, low density lipoproteins, and triglycerides was observed. Their results suggested that the combination of HCA-chromium-NBC may be an effective formula for losing weight and promoting healthy blood lipid levels.	[36]
2009	Carvajal-Zarrabal et al.	The objective was to analyze the effect of three ethanolic extracts of Hs dry calyx on fat absorption-excretion and body weight in Sprague-Dawley rats. One group of animals was fed a normal diet and others with the same diet plus the supplement of each extract [5% (Hs5), 10% (Hs10) and 15% (Hs15)]. Only Hs5 showed no changes in weight and food consumption; unlike the group treated with Hs15 where there was a significant decrease in these parameters and a greater amount of fatty acids found in the feces. It was concluded that this anti-obesity effect is possibly attributed to the presence of the HCA from the extracts.	[26]
2012	Márquez et al.	Information was collected from different authors confirming that the administration of G. cambogia extracts (where HCA is found) is associated with weight reduction and fat loss. However, these Spanish researchers suggest to be cautious when interpreting the results, as there are conflicting data in some randomized and controlled clinical trials. In addition, they indicate that the majority of human studies have been conducted in small samples and mainly in the short term. None of them have demonstrated whether these effects persist beyond 12 weeks of intervention. There is little evidence on the long-term benefits of extracts, and especially HCA. Regarding its toxicity, they suggest considering the evidence that HCA can cause acute skin lesions if applied directly to the skin, cause eye irritation and increase the level of peripheral testosterone by stimulating erythropoiesis in humans. Also, it is important to consider that, during fetal development, there is a high demand for lipid production for growth and that the lipids produced by the mother are transferred through the placenta to the fetus; therefore, possibly the HCA by inhibiting the synthesis and storage of lipids may be critical for gestational development.	[41]
2016	Goudarzvand et al.	The study evaluated the administration of HCA (2 g/kg/day for 3 weeks) in C57BL/6 mice with multiple sclerosis. At the end of the period the treatment improved the symptoms of nerve injury, decreasing the levels of serum interleukin-6 (IL-6), TNF-α, MDA, and nitric oxide (NO). It was also observed that the activity of superoxide dismutase (SOD) and glutathione reductase (GR) increased; in consequence, HCA may have neuroprotective effects through anti-inflammatory and antioxidant mechanisms	[42]
2019	Han et al.	To assess the effects of the long-term supplement with HCA on weight gain and variations in amino acid content in rats. Significant loss of body weight and increased thyroid hormone levels were observed. These results suggest that HCA promotes energy expenditure by regulating thyroid hormone levels. In addition, it possibly stimulates protein synthesis by altering the metabolic directions of amino acids.	[43]
2019	Ibuki et al.	It is known that age-related macular degeneration (AMD) is the leading cause of blindness. Likewise, different evidence have established that the anti-vascular endothelial growth factor (VEGF) therapy has a potent therapeutic effect against the disease. There is the possibility of presenting different systemic adverse events, such as chorio-retinal atrophy, due to the long-term antagonism of VEGF. The objective of this study was to explore the effect of HCA on the hypoxia-inducible factor (HIF) regulation of VEGF transcription. At the end of the study, it was confirmed that HCA showed an inhibitory effect on HIF in the luciferase assay.	[44]
2019	Tomar et al.	It was a clinical and computational study (100 individuals under a 3-month treatment) on the anti-obesity effects of HCA. Anthropometric parameters and plasma lipid profiles were evaluated. A hepatic metabolic model was used to incorporate the effect of HCA at the level of the metabolic pathway. In addition, the activity of ATP citrate lyase in the metabolic pathway was analyzed to simulate the net effect of HCA. The results showed a reduction in the synthesis of fatty acids, triglycerides and cholesterol. It was concluded that treatment with HCA can reduce body weight gain and fat accumulation in obese subjects along with the improvement of their anthropometric parameters and metabolic status.	[45]

## References

[1] Patel S. (2014). *Hibiscus sabdariffa*: An Ideal yet under-exploited candidate for nutraceutical applications. Biomed. Prev. Nutr..

[2] Rafael A.-F., Víctor S.-A., Salvador N.-G., Manuel E.O.-C., Enrique V.-G., Barrios-Ayala A., Michel-Aceves A.C., Guzmán-Maldonado S.H., Otero-Sánchez M.A. (2014). Variedades mexicanas de jamaica (*Hibiscus sabdariffa L.*) ‘alma blanca’ y ‘rosalíz’ de color claro, y ‘cotzaltzin’ y ‘tecoanapa’ de color rojo. Rev. Fitotec. Mex..

[3] Da-Costa-Rocha I., Bonnlaender B., Sievers H., Pischel I., Heinrich M. (2014). *Hibiscus sabdariffa* L. A phytochemical and pharmacological review. Food Chem..

[4] Riaz G., Chopra R. (2018). A review on phytochemistry and therapeutic uses of *Hibiscus sabdariffa* L.. Biomed. Pharmacother..

[5] Ojulari O.V., Lee S.G., Nam J.O. (2019). Beneficial Effects of Natural Bioactive Compounds from *Hibiscus sabdariffa* L. on obesity. Molecules.

[6] Najafpour Boushehri S., Karimbeiki R., Ghasempour S., Ghalishourani S.S., Pourmasoumi M., Hadi A., Mbabazi M., Pour Z.K., Assarroudi M., Mahmoodi M. (2020). The efficacy of sour tea (*Hibiscus sabdariffa* L.) on selected cardiovascular disease risk factors: A systematic review and meta-analysis of randomized clinical trials. Phytother. Res..

[7] Serban C., Sahebkar A., Ursoniu S., Andrica F., Banach M. (2015). Effect of sour tea (*Hibiscus sabdariffa* L.) on arterial hypertension: A systematic review and meta-analysis of randomized controlled trials. J. Hypertens..

[8] Gheller A.C.G.V., Kerkhoff J., Vieira Júnior G.M., Campos K.E., Sugui M.M. (2017). Antimutagenic Effect of *Hibiscus sabdariffa* L. Aqueous Extract on Rats Treated with Monosodium Glutamate. Sci. World J..

[9] Akindahunsi A.A., Olaleye M.T. (2003). Toxicological investigation of aqueous-methanolic extract of the calyces of *Hibiscus sabdariffa* L.. J. Ethnopharmacol..

[10] Bule M., Albelbeisi A.H., Nikfar S., Amini M., Abdollahi M. (2020). The antidiabetic and antilipidemic effects of *Hibiscus sabdariffa*: A systematic review and meta-analysis of randomized clinical trials. Food Res. Int..

[11] Zhang B., Yue R., Wang Y., Wang L., Chin J., Huang X., Jiang Y. (2019). Effect of *Hibiscus sabdariffa* (Roselle) supplementation in regulating blood lipids among patients with metabolic syndrome and related disorders: A systematic review and meta-analysis. Phytother. Res..

[12] Aziz Z., Wong S.Y., Chong N.J. (2013). Effects of *Hibiscus sabdariffa* L. on serum lipids: A systematic review and meta-analysis. J. Ethnopharmacol..

[13] Hassan S.T., Berchova K., Sudomova M. (2016). Antimicrobial, antiparasitic and anticancer properties of *Hibiscus sabdariffa* (L.) and its phytochemicals: In vitro and in vivo studies. Ceska Slov. Farm..

[14] Sumaya Martínez M.T., Medina-Carrillo R.E., Machuca-Sánchez M.L., Jiménez-Ruiz E., Balois- Morales R., Sánchez- Herrera L.M. (2014). Potencial de la jamaica (*Hibiscus sabdariffa* L.) en la elaboración de alimentos funcionales con actividad antioxidante. Rev. Mex. de Agronegocios.

[15] Cid-Ortega S., Guerrero-Beltrán J.A. (2012). Propiedades funcionales de la Jamaica (*Hibiscus sabdariffa* L). Temas Selectos de Ingeniería en Alimentos.

[16] Carvajal-Zarrabal O., Barradas-Dermitz D.M., Orta-Flores Z., Hayward-Jones P.M., Nolasco-Hipolito C., Aguilar-Uscanga M.G., Miranda-Medina A., Bujang K.B. (2012). *Hibiscus sabdariffa* L., roselle calyx, from ethnobotany to pharmacology. J. Exp. Pharmacol..

[17] Guardiola S., Mach N. (2014). Therapeutic potential of *Hibiscus sabdariffa*: A review of the scientific evidence. Endocrinol. Nutr..

[18] Herranz-Lopez M., Olivares-Vicente M., Encinar J.A., Barrajon-Catalan E., Segura-Carretero A., Joven J., Micol V. (2017). Multi-Targeted Molecular Effects of Hibiscus sabdariffa Polyphenols: An Opportunity for a Global Approach to Obesity. Nutrients.

[19] Hopkins A.L., Lamm M.G., Funk J.L., Ritenbaugh C. (2013). *Hibiscus sabdariffa* L. in the treatment of hypertension and hyperlipidemia: A comprehensive review of animal and human studies. Fitoterapia.

[20] McKay D. (2019). Can hibiscus tea lower blood pressure?. Afro Food Ind. Hi-Tech.

[21] Fakeye T. (2008). Toxicity and immunomodulatory activity of *Hibiscus sabdariffa* Linn (Family Malvaceae) in animal models. Afr. J. Tradit. Complement. Altern. Med..

[22] Kao E.S., Hsu J.D., Wang C.J., Yang S.H., Cheng S.Y., Lee H. (2009). Polyphenols extracted from *Hibiscus sabdariffa* L. inhibited lipopolysaccharide induced inflammation by improving antioxidative conditions and regulating cyclooxygenase-2 expression. Biosci. Biotechnol. Biochem..

[23] Josiah S.J., Omotuyi O., Oluyemi K.A., Ezea U.I., Uhunmwangho E.S., Nwangwu C.O. (2010). Protective role of aqueous extract of *Hibiscus sabdariffa* (calyx) against potassium bromate induced tissue damage in Wistar rats. Afr. J. Biotechnol..

[24] Tseng T.H., Wang C.J., Kao E.S., Chu H.Y. (1996). Hibiscus protocatechuic acid protects against oxidative damage induced by tert-butylhydroperoxide in rat primary hepatocytes. Chem. Biol. Interact..

[25] Lo C.W., Huang H.P., Lin H.M., Chien C.T., Wang C.J. (2007). Effect of Hibiscus anthocyanins-rich extract induces apoptosis of proliferating smooth muscle cell via activation of P38 MAPK and p53 pathway. Mol. Nutr. Food Res..

[26] Carvajal-Zarrabal O., Hayward J.P.M., Orta F.Z., Nolasco H.C., Barradas D.D.M., Aguilar U.M.G., Pedroza H.M.F. (2009). Effect of *Hibiscus sabdariffa* L. dried calyx ethanol extract on fat absorption-excretion, and body weight implication in rats. J. Biomed. Biotechnol..

[27] Ali B.H., Al Wabel N., Blunden G. (2005). Phytochemical, pharmacological and toxicological aspects of Hibiscus sabdariffa L.: A review. Phytother. Res..

[28] Abubakar M.G., Lawal A., Sulelman B., Abdullahl K. (2010). Hepatorenal toxicity stuies of sub-chronic administration of calyx aqueous extracts of Hibiscus sabdariffa in albino rats. Bayero J. Pure Appl. Sci..

[29] Mahmoud Y.I. (2012). Effect of extract of hibiscus on the ultrastructure of testis in adult mice. Acta Histochem..

[30] Johnson S.S., Oyelola F.T., Ari T., Juho H. (2013). In vitro inhibitory activities of the extract of Hibiscus sabdariffa L. (family Malvaceae) on selected cytochrome P450 isoforms. Afr. J. Tradit. Complement. Altern. Med..

[31] Salem M.Z.M., Olivares-Pérez J., Salem A.Z.M. (2014). Studies on biological activities and phytochemicals composition of *Hibiscus* species-A review. Life Sci. J..

[32] Chiu C.T., Chen J.H., Chou F.P., Lin H.H. (2015). *Hibiscus sabdariffa* leaf extract inhibits human prostate cancer cell invasion via down-regulation of Akt/NF-kB/MMP-9 pathway. Nutrients.

[33] Lin H.H., Chen J.H., Kuo W.H., Wang C.J. (2007). Chemopreventive properties of *Hibiscus sabdariffa* L. on human gastric carcinoma cells through apoptosis induction and JNK/p38 MAPK signaling activation. Chem. Biol. Interact..

[34] Malacrida A., Maggioni D., Cassetti A., Nicolini G., Cavaletti G., Miloso M. (2016). Antitumoral effect of *Hibiscus sabdariffa* on human squamous cell carcinoma and multiple myeloma cells. Nutr. Cancer.

[35] Lin H.H., Chen J.H., Wang C.J. (2011). Chemopreventive properties and molecular mechanisms of the bioactive compounds in *Hibiscus sabdariffa* Linne. Curr. Med. Chem..

[36] Preuss H.G., Echard B., Bagchi D., Stohs S. (2007). Inhibition by Natural Dietary Substances of Gastrointestinal Absorption of Starch and Sucrose in Rats 2. Subchronic Studies. Int. J. Med. Sci..

[37] Zheoat A.M., Gray A.I., Igoli J.O., Ferro V.A., Drummond R.M. (2019). Hibiscus acid from *Hibiscus sabdariffa* (Malvaceae) has a vasorelaxant effect on the rat aorta. Fitoterapia.

[38] Hansawasdi C., Kawabata J., Kasai T. (2000). Alpha-amylase inhibitors from roselle (*Hibiscus sabdariffa* Linn.) tea. Biosci. Biotechnol. Biochem..

[39] Hansawasdi C., Kawabata J., Kasai T. (2001). Hibiscus acid as an inhibitor of starch digestion in the Caco-2 cell model system. Biosci. Biotechnol. Biochem..

[40] Hayamizu K., Ishii Y., Kaneko I., Shen M., Okuhara Y., Shigematsu N., Tomi H., Furuse M., Yoshino G., Shimasaki H. (2003). Effects of *Garcinia cambogia* (Hydroxycitric Acid) on visceral fat accumulation: A double-blind, randomized, placebo-controlled trial. Curr. Ther. Res. Clin. Exp..

[41] Márquez F., Babio N., Bulló M., Salas-Salvadó J. (2012). Evaluation of the safety and efficacy of hydroxycitric acid or *Garcinia cambogia* extracts in humans. Crit. Rev. Food Sci. Nutr..

[42] Goudarzvand M., Afraei S., Yaslianifard S., Ghiasy S., Sadri G., Kalvandi M., Alinia T., Mohebbi A., Yazdani R., Azarian S.K. (2016). Hydroxycitric acid ameliorates inflammation and oxidative stress in mouse models of multiple sclerosis. Neural. Regen. Res..

[43] Han N., Li L., Peng M., Ma H. (2019). (−)-Hydroxycitric Acid Nourishes Protein Synthesis via Altering Metabolic Directions of Amino Acids in Male Rats. Phytother. Res..

[44] Ibuki M., Shoda C., Miwa Y., Ishida A., Tsubota K., Kurihara T. (2019). Therapeutic Effect of *Garcinia cambogia* Extract and Hydroxycitric Acid Inhibiting Hypoxia-Inducible Factor in a Murine Model of Age-Related Macular Degeneration. Int. J. Mol. Sci..

[45] Tomar M., Rao R.P., Dorairaj P., Koshta A., Suresh S., Rafiq M., Venkatesh K.V. (2019). A clinical and computational study on anti-obesity effects of hydroxycitric acid. RSC Adv..

[46] Gabutti L., Ferrari N., Mombelli G., Keller F., Marone C. (2004). The favorable effect of regional citrate anticoagulation on interleukin-1beta release is dissociated from both coagulation and complement activation. J. Nephrol..

[47] Abdel-Salam O.M.E., Youness E.R., Mohammed N.A., Youssef M.S.M., Omara E.A., Sleem A.A. (2014). Citric Acid Effects on Brain and Liver Oxidative Stress in Lipopolysaccharide-Treated Mice. J. Med. Food.

[48] Kornhauser A., Coelho S.G., Hearing V.J. (2012). Effects of Cosmetic Formulations Containing Hydroxyacids on Sun-Exposed Skin: Current Applications and Future Developments. Dermatol. Res. Pract..

[49] Tang X., Liu J., Dong W., Li P., Li L., Lin C., Zheng Y., Hou J., Li D. (2013). The Cardioprotective Effects of Citric Acid and L-Malic Acid on Myocardial Ischemia/Reperfusion Injury. Evid. Based Complement. Altern. Med..

[50] Gómez-Moreno G., Guardia J., Aguilar S.A., Cabrera A.M., Maté-Sánchez V.J.E., Calvo G.J.L. (2013). Effectiveness of malic acid 1% in patients with xerostomia induced by antihypertensive drugs. Med. Oral Patol. Oral Cir. Bucal..

[51] Hsiao Y.P., Lai W.W., Wu S.B., Tsai C.H., Tang S.C., Chung J.G., Yang J.H. (2015). Triggering Apoptotic Death of Human Epidermal Keratinocytes by Malic Acid: Involvement of Endoplasmic Reticulum Stress-and Mitochondria-Dependent Signaling Pathways. Toxins.

[52] Xuan J., Feng Y. (2019). Enantiomeric Tartaric Acid Production Using cis-Epoxysuccinate Hydrolase: History and Perspectives. Molecules.

[53] Du J., Cullen J.J., Buettner G.R. (2012). Ascorbic acid: Chemistry, biology and the treatment of cancer. Biochim. Biophys. Acta.

[54] Lachapelle M.Y., Drouin G. (2011). Inactivation dates of the human and guinea pig vitamin C genes. Genetica.

[55] Covarrubias-Pinto A., Acuña A.I., Beltrán F.A., Torres-Díaz L., Castro M.A. (2015). Old Things New View: Ascorbic Acid Protects the Brain in Neurodegenerative Disorders. Int. J. Mol. Sci..

[56] Williamson E. (2009). Stockley’s Herbal Medicines Interactions: A Guide to the Interactions of Herbal Medicines, Dietary Supplements and Nutraceuticals with Conventional Medicines.

[57] Lin H.H., Huang H.P., Huang C.C., Chen J.H., Wang C.J. (2005). Hibiscus polyphenol-rich extract induces apoptosis in human gastric carcinoma cells via p53 phosphorylation and p38 MAPK/FasL cascade pathway. Mol. Carcinog..

[58] Yamamoto R., Osima Y. (1932). On the red colouring matter of *Hibiscus sabdariffa* L. (A new glycoside hiviscin). J. Agric. Chem. Soc. Jpn..

[59] Zheoat A.M., Gray A.I., Igoli J.O., Kennedy A.R., Ferro V.A. (2017). Ferro Crystal structures of hibiscus acid and hibiscus acid dimethyl ester isolated from *Hibiscus sabdariffa* (Malvaceae). Acta Crystallogr. E Crystallogr. Commun..

[60] Aidé S.G., Alejandra M.V., Lluvia L., Liliana C.F., Leticia B.B. (2014). Ácido Cítrico: Compuesto Interesante. Rev. Cient. Univ. Auton. Coahuila.

[61] Hu W., Li W.J., Yang H.Q., Chen J.H. (2019). Current strategies and future prospects for enhancing microbial production of citric acid. Appl. Microbiol. Biotechnol..

[62] Ramesh T., Kalaiselvam M. (2011). An Experimental Study on Citric Acid Production by *Aspergillus niger* Using Gelidiella acerosa as a Substrate. Indian J. Microbiol..

[63] Poerwono H., Higashiyama K., Kubo H., Poernomo A.T., Brittain H.G., Brittain Harry G. (2001). Citric Acid. Analytical Profiles of Drug Substances and Excipients.

[64] Vandenberghe L.P.S., Soccol C.R., Pandey A., Lebeault J.M. (1999). Microbial production of citric acid. Braz. Arch. Boil. Technol..

[65] Pimenta F.C.F., Cunha-Tavares N.A., Chaves-Neto G., Alves M., Fernandes-Pimenta M., Melo-Diniz J., Correia de Medeiros A., Melo-Diniz M.F.F., Gill H., Garg H. (2017). Pharmacological Actions of Citrus Species. Citrus Pathology.

[66] Doizi S., Poindexter J.R., Pearle M.S., Blanco F., Moe O.W., Sakhaee K., Maalouf N.M. (2018). Impact of Potassium Citrate vs. Citric Acid on Urinary Stone Risk in Calcium Phosphate Stone Formers. J. Urol..

[67] Yamada T., Hida H., Yamada Y. (2007). Chemistry, physiological properties, and microbial production of hydroxycitric acid. Appl. Microbiol. Biotechnol..

[68] Peng M., Han J., Li L., Ma H. (2016). Suppression of fat deposition in broiler chickens by (−)-hydroxycitric acid supplementation: A proteomics perspective. Sci. Rep..

[69] Portillo-Torres L.A., Bernardino-Nicanor A., Gómez-Aldapa C.A., González-Montiel S., Rangel-Vargas E., Villagómez-Ibarra J.R., González-Cruz L., Cortés-López H., Castro-Rosas J. (2019). Hibiscus Acid and Chromatographic Fractions from *Hibiscus sabdariffa* Calyces: Antimicrobial Activity against Multidrug-Resistant Pathogenic Bacteria. Antibiotics.

[70] Madrigal-Santillán E., Sánchez-Gutiérrez M., Izquierdo-Vega J.A., Valadez-Vega M.C., Gómez-Aldapa C., Castro-Rosas J. Assessment of the genotoxic potential of a methanolic extract from roselle (*Hibiscus sabdariffa* L.) in vivo. Proceedings of the XXVIII Italo-Latin American Ethnomedicine Congress (SILAE).

[71] Morales-González J.A., Madrigal-Bujaidar E., Sánchez-Gutiérrez M., Izquierdo-Vega J.A., Valadez-Vega M.D.C., Álvarez-González I., Morales-González Á., Madrigal-Santillán E. (2019). Garlic (*Allium sativum* L.): A Brief Review of Its Antigenotoxic Effects. Foods.

